# Association of supermarket characteristics with the body mass index of their shoppers

**DOI:** 10.1186/1475-2891-12-117

**Published:** 2013-08-13

**Authors:** Scott A Lear, Danijela Gasevic, Nadine Schuurman

**Affiliations:** 1 Faculty of Health Sciences, Simon Fraser University, 8888 University Drive, Burnaby V5A 1S6British Columbia, Canada; 2 Department of Biomedical Physiology and Kinesiology, Simon Fraser University, 515 West Hastings Street, Vancouver, British Columbia V6B 5K3, Canada; 3 Division of Cardiology, Providence Health Care, 1081 Burrard Street, Vancouver, British Columbia V6Z 1Y6, Canada; 4 Department of Geography, Simon Fraser University, 8888 University Drive, Burnaby V5A 1S6British Columbia, Canada

**Keywords:** Supermarket, Shoppers, Body mass index, Obesity, Food basket

## Abstract

**Background:**

Research on the built food environment and weight status has mostly focused on the presence/absence of food outlets while ignoring their internal features or where residents actually shop. We explored associations of distance travelled to supermarkets and supermarket characteristics with shoppers’ body mass index (BMI).

**Methods:**

Shoppers (n=555) of five supermarkets situated in different income areas in the city were surveyed for food shopping habits, demographics, home postal code, height and weight. Associations of minimum distance to a supermarket (along road network, objectively measured using ArcGIS), its size, food variety and food basket price with shoppers’ BMI were investigated. The ‘food basket’ was defined as the mixture of several food items commonly consumed by residents and available in all supermarkets.

**Results:**

Supermarkets ranged in total floor space (7500–135 000 square feet) and had similar varieties of fruits, vegetables and cereals. The majority of participants shopped at the surveyed supermarket more than once per week (mean range 1.2 ± 0.8 to 2.3 ± 2.1 times per week across the five supermarkets, p < 0.001), and identified it as their primary store for food (52% overall). Mean participant BMI of the five supermarkets ranged from 23.7 ± 4.3 kg/m^2^ to 27.1 ± 4.3 kg/m^2^ (p < 0.001). Median minimum distance from the shoppers’ residence to the supermarket they shopped at ranged from 0.96 (0.57, 2.31) km to 4.30 (2.83, 5.75) km (p < 0.001). A negative association was found between food basket price and BMI. There were no associations between BMI and minimum distance to the supermarket, or other supermarket characteristics. After adjusting for age, sex, dissemination area median individual income and car ownership, BMI of individuals who shopped at Store 1 and Store 2, the supermarkets with lowest price of the ‘food basket’, was 3.66 kg/m^2^ and 3.73 kg/m^2^ higher compared to their counterparts who shopped at the supermarket where the ‘food basket’ price was highest (p < 0.001).

**Conclusions:**

The food basket price in supermarkets was inversely associated with BMI of their shoppers. Our results suggest that careful manipulation of food prices may be used as an intervention for decreasing BMI.

## Introduction

At a fundamental level, obesity is predominantly the result of a positive caloric imbalance from more calories consumed than expended. Given the recent and unabated rise in the prevalence of obesity [1], much research has been focused on identifying key determinants that may be acted upon for future prevention and intervention strategies. Of these possible determinants, the environment in which we live has gained considerable attention [2,3]. Specifically, the built environment (BE, the human-made or modified environmental surroundings) has been implicated in the recent rapid rise in obesity throughout the world [4].

A number of studies have reported associations between a variety of BE features and indicators of obesity. Most of these studies have focused on features of the environment that may act as either barriers or facilitators of physical activity (and predominantly walking) [5-13]. Comparatively, fewer studies have investigated the link between food availability in the environment and obesity. These studies have indicated that obesity rates tend to be higher in low income areas [14], where there is a higher density of fast-food restaurants and less access to fruits, vegetables and other healthy foods [15-18]. Conversely, residents of neighbourhoods that have a greater number of supermarkets have more nutritious diets [19] and lower obesity rates [20].

Many of these studies investigating supermarkets and other food outlets have been limited to assessing their presence or absence within a defined environment or ‘neighbourhood’. For these investigations, a neighbourhood was commonly defined as a circular radius around each individual [19,21,22], a buffer around the center point of the census tract [23], or it was solely based on the administrative units such as census tracts [20,21,24-26]. An underlying assumption in these studies is that people predominantly access food within the neighbourhood boundaries. While these studies have been instrumental in furthering our understanding of how the environment is associated with obesity, they did not take into account the possibility that people may travel outside of their neighbourhood to access food. In addition, only a few studies have investigated the relationship between supermarket characteristics and obesity. These early studies have observed a positive relationship between the availability of nutritious foods in supermarkets and the foods in the diets of nearby residents, suggesting that certain supermarket characteristics may be related to dietary intake and in turn obesity [25,27]. As the majority of people still frequent supermarkets for their food shopping [28], supermarkets may be a potential point of intervention for obesity prevention, however, gaps exist in our understanding of the association between food availability, accessibility and obesity. Therefore, the purpose of this investigation was to identify associations of minimum distance from shoppers’ residence to supermarkets and their characteristics (variety of foods, size and price) with shoppers’ body mass index (BMI).

## Methods and procedures

A total of five supermarkets were identified within the City of Vancouver, British Columbia, Canada. These supermarkets were purposely selected to represent a range of sizes (7000 – 135 000 square feet) in different income areas in the city. These five supermarkets came from four supermarket chains that had several locations throughout the Greater Vancouver area. Each store was assessed for the price of a ‘food basket’ prior to initiation of the shopper survey. The ‘food basket’ was defined as the mixture of the following food items (unit of purchase): 2 percent fat milk (4L), bananas (per lb), tomatoes (per lb), eggs (medium size, dozen), white rice (900 grams), white flour (2.5 kg), white sugar (1 kg) and white bread (loaf). These foods were selected as they were available in all five supermarkets and are commonly consumed. In situations where more than one food type or brand was available, the cheapest product was priced, which was commonly the supermarket’s own brand. Furthermore, the number of available different fruits, vegetables and cereals was obtained by a research assistant counting the varieties from each store. The supermarket’s dimensions were provided by the supermarket manager.

On separate weeks, each supermarket was observed near the main exit of the store to count the number of individuals leaving the store during daytime hours (9:00 to 16:00) for one weekday and the following Saturday. The total number of individuals as well as the apparent sex of the individuals was determined. These numbers were used to identify the total number of potential study participants. The following week two trained research assistants stood outside the exit of each supermarket and invited consecutive individuals leaving the supermarket to complete a health-related survey. However, given the number of shoppers leaving the supermarkets, not every person was approached such as when both research assistants were conducting surveys with shoppers. The surveys were conducted on the same days and time as the observations in the previous week (a Thursday and a Saturday). The survey was designed to be brief (less than 5 minutes) in recognition of a limited window of time to be administered as individuals exited the supermarket with their groceries. The survey included questions on individual food shopping habits (frequency of shopping, primary household shopper, whether the current supermarket was their main shopping source for food, current and monthly grocery bill, wholesale shopping), demographics (age, sex, postal code, car ownership- the latter as a proxy of income), mode of transportation to the supermarket, blocks to the nearest produce store from their residence and self- reported height and weight to calculate BMI. The survey was first pilot tested in two other supermarkets not included in the analysis prior to initiation. This study was approved by the Simon Fraser University Research Ethics Board.

Of the 555 survey participants, 516 participants provided full six digit postal codes. These participant postal codes were geocoded in ArcMap using the CanMap Postal Geography 2006(v3) Unique Enhanced Postal Codes by DMTI for the locator file. Of the 516 customer entries, 17 postal codes were unmatched. It was found that two of the unmatched entries had associated addresses, and using canadapost.ca, the postal codes were found, so they were added. The other 15 were excluded. There were also six outliers which geocoded to locations several hours drive outside of the Vancouver area. Three of these appeared to be visitors who did not frequent the supermarkets regularly and three appeared to be mistakes. All six were excluded from analysis.

Supermarket addresses were geocoded in ArcMap using the Road Atlas of British Columbia by GIS Innovations for the locator file. ArcCatalog and Network Analyst tools were used to create a geodatabase road network dataset from the Road Atlas of British Columbia file. Before building the network dataset, restricted road segments were removed since they are not generally accessible to the general public and only segments in the Greater Vancouver Regional District and Fraser Valley Regional District were used in order to limit the size of the file. Road network attributes were created for travel distance and permitted direction of travel on a road. All spatial data processing and analysis was done using ESRI ArcGIS 9.2 software. The Network Analyst Closest Facility tool was used to create a route map to each supermarket from all the customer participants’ locations. The road network dataset was used along with the supermarket and customer locations which had been grouped by supermarket.

In order to obtain residential area income levels of the participants, average household annual income data were obtained for the participant’s residing census dissemination area. To do so, a British Columbia 2001 (this was the latest year for available data that coincided with study data collection) Census Dissemination Area coverage map and median total individual income values for British Columbia were obtained from Statistics Canada. They were joined using the DAUID field in the Dissemination Area coverage table and a concatenation of the CDCode and DisseminationArea fields in the income data. The data were then limited to entries with a CDName of Greater Vancouver Regional District or Fraser Valley Regional District. Sets were also created for the customers of each store using selection by location where the saved Dissemination Areas “contained” a customer location point. These data were plotted using chloropleth maps having different colours for the income levels of the dissemination areas.

### Statistical analyses

Continuous variables are reported as means ± standard deviations, and categorical variables as percentages and counts. Evaluation of inter- supermarket differences was carried out using ANOVA for continuous variables and the Chi-square test for categorical variables. Pearson correlation coefficients were determined to assess the association between supermarket characteristics and the mean BMI of the store participants. Supermarket characteristics showing significant correlations with BMI were further carried to multiple linear regression analysis to explore their associations with BMI after adjusting for age (continuous), sex (males, females), residential dissemination area median individual income (continuous) and personal car ownership (yes, no). The latter two variables were included as proxies of participant socio-economic status. Analyses were performed on 423 participants in whom we had complete data. Data were analyzed using PASW Statistics 18.0 software. The level of significance was set at 0.05.

## Results

Customer participation rates were calculated based on the number of customers participating in the survey divided by the number of customers observed on the previous observation days, and ranged across the supermarkets from 14% to 28% (Table 1). The five stores ranged in total floor space from 7500 square feet to 135 000 square feet and had similar varieties of fruits, vegetables and cereals (Table 1).

**Table 1:** Store characteristics

	**Store 1**	**Store 2**	**Store 3**	**Store 4**	**Store 5**
Customers observed during observation days	4185	1516	1570	2084	2195
Customers participating	77	90	135	153	100
Average household income area	$62 692	$38 141	$26 289	$47 015	$65 859
Floor space of store (square feet)	135 000	7500	28 000	25 000	43 000
Food basket price	$15.46	$16.75	$17.35	$18.96	$20.60
Variety of fruits (count)	47	34	46	48	39
Variety of vegetables (count)	83	93	80	81	79
Variety of cereals (count)	119	106	98	84	91

**Figure 1:**
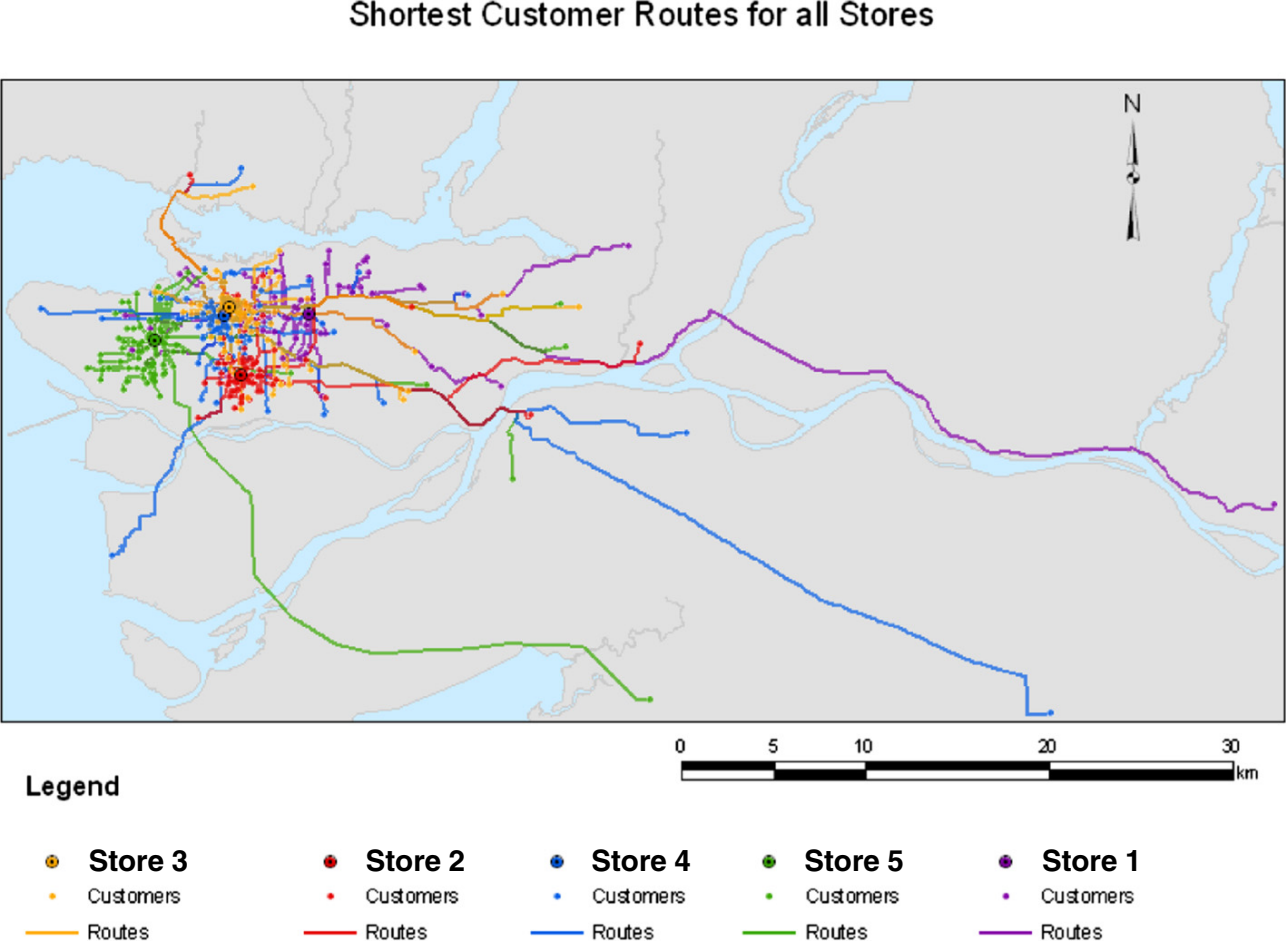
Minimum road travel routes between the store and the residential postal code of the store participants.

Table 2 outlines the customer participant characteristics by supermarket. There were significant differences in all assessed characteristics across the five supermarkets except for the proportion of participants being the primary shopper. The majority of participants shopped at the surveyed supermarket more than once per week (mean range 1.2 ± 0.8 to 2.3 ± 2.1 times per week across the stores, p < 0.001), was the designated primary food shopper of their household (range from 70.0% to 83.1% across the supermarkets) and identified it as their primary store for food (52% overall). Mean participant BMI ranged from 23.7 ± 4.3 kg/m^2^ to 27.1 ± 4.3 kg/m^2^ (p < 0.001). In addition, median minimum distance shoppers their residence to the supermarket they shopped at ranged from 0.96 (0.57, 2.31) km to 4.30 (2.83, 5.75) km (p < 0.001) (Table 2). These minimum road travel routes between the supermarket and the participants’ residential postal code are displayed on Figure 1. Participant transportation to the supermarket differed across the stores such that more participants reported walking to the supermarkets that were closer to their place of residence (p < 0.001).

**Table 2:** Participant demographics, shopping habits, lifestyle and anthropometry stratified by store

	**Store 1**	**Store 2**	**Store 3**	**Store 4**	**Store 5**
	**n=77**	**n=90**	**n=135**	**n=153**	**n=100**
Age (years)	45.4 ± 15.9	49.7 ± 16.8	44.4 ± 14.4	47.4 ± 17.3	55.7 ± 20.2**
Women (%)	42 (54.5%)	42 (47.2%)	63 (46.7%)	81 (52.9%)	73 (73.0%)++
Car ownership	70 (90.9%)	62 (68.9%)	69 (51.1%)	108 (70.6%)	83 (83.0%)+++
Residential area income ($)†	$24 527 ($22 303, $28 448)	$22812 ($20 560, $25 825)	$23334 ($20 908, $27 235)	$28962 ($22 446, $33 215)	$45 595 ($35 659, $56 529)**
Minimum distance to store (km)†‡	4.30 (2.83, 5.75)	1.32 (0.72, 1.96)	0.96 (0.57, 2.31)	1.23 (0.71, 2.64)	2.29 (1.50, 3.28)**
Transportation to store					
Car	70 (90.9%)	41 (45.6%)	48 (35.5%)	81 (52.9%)	82 (82.0%)+++
Transit	4 (5.2%)	13 (14.4%)	18 (13.3%)	4 (2.6%)	7 (7.0%)
Walking	2 (2.6%)	32 (35.6%)	55 (40.7%)	57 (37.3%)	8 (8.0%)
Bicycle	0 (0%)	3 (3.3%)	12 (8.9%)	8 (5.2%)	0 (0%)
Frequency of shopping at this supermarket (per week)	1.2 ± 0.8	2.3 ± 2.1	1.9 ± 1.7	1.7 ± 1.8	1.6 ± 1.4**
Primary store for food shopping (%)	51 (66.2%)	46 (51.1%)	71 (52.6%)	55 (35.9%)	63 (63.0%)+++
Primary shopper in household (%)	64 (83.1%)	63 (70.0%)	111 (82.2%)	123 (80.4%)	80 (80.0%)
Grocery bill today	$69.28 ± $66.25	$21.54 ± $24.26	$17.50 ± $26.35	$21.19 ± $26.45	$53.73 ± $48.43**
Distance to nearest produce store (blocks)†	5 (2, 7)	3 (2, 5)	3 (2, 5)	4 (2, 7)	4 (2, 6)**
Body mass index (kg/m^2^)	27.1 ± 4.3	27.6 ± 4.6	25.4 ± 4.7	25.1 ± 4.7	23.7 ± 4.3**

Categorical data presented as n(%). Continuous normally distributed variables presented as Mean ± SD, while variables not following normal distribution (indicated by †) presented as Median (25%, 75%).

ANOVA for overall comparison across stores: * p<0.05, ** p<0.001; Pearson Chi-square test: + p<0.05, ++ p<0.01, +++ p<0.001; ‡ assessed objectively using GIS; the rest of the variables self-reported.

**Figure 2:**
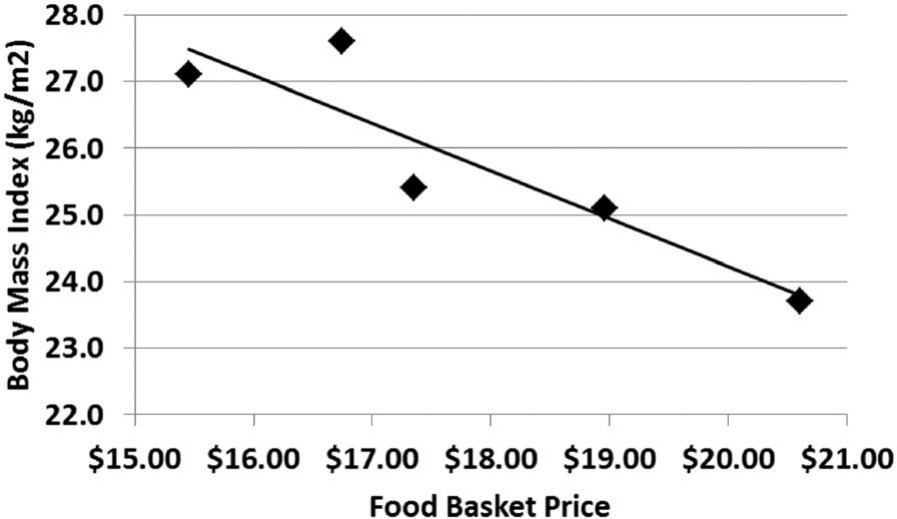
Relationship between store mean participant body mass index and food basket price.

The cost of the food basket was negatively correlated with mean participant BMI (r=−0.906, p=0.034) (Figure 2). However, there were no correlations between mean BMI and median distance to the supermarket, or with any other supermarket characteristics such as floor space of supermarkets, variety of fruits, vegetables and cereals (Table 3). Even after adjusting for age, sex, dissemination area median individual income and personal car ownership, an increase in the supermarket food basket price was associated with decrease in BMI of supermarket shoppers (Table 4). Namely, BMI of individuals who shopped at Store 1 and Store 2, the supermarkets with lowest price of the ‘food basket’, was 3.66 kg/m^2^ and 3.73 kg/m^2^ higher compared to their counterparts who shopped at the supermarket where the ‘food basket’ price was highest (p < 0.001). In addition, individuals who shopped at Store 3 in which mean food basket price was $17.35 had BMI higher by 1.93 kg/m^2^ than residents who shopped in a supermarket with the highest price ($20.60) of a food basket (p = 0.029).

**Table 3:** Results of Pearson correlations between mean participant body mass index and store characteristics

	**Pearson correlation coefficient**	**p value**
Food basket price	−0.906	0.034
Floor space of store	0.235	0.704
Median minimum distance to store	0.128	0.837
Variety of fruits	−0.191	0.758
Variety of vegetables	0.809	0.097
Variety of cereals	0.773	0.125

**Table 4:** Association between food basket price and BMI

	**Beta coefficient (Standard Error)**	**p value**
Store (food basket price)*		
Store 1 ($15.46) vs. Store 5 ($20.60)	3.66 (0.94)	< 0.001
Store 2 ($16.75) vs. Store 5 ($20.60)	3.73 (0.94)	< 0.001
Store 3 ($17.35) vs. Store 5 ($20.60)	1.93 (0.88)	0.029
Store 4 ($18.96) vs. Store 5 ($20.60)	1.52 (0.80)	0.057

Association between food basket price (independent variable) and BMI (dependent variable) was explored by multiple linear regression. The model was adjusted for age, sex, residential area median income and personal car ownership.

* Store 1, lowest food basket price; Store 5, highest food basket price.

## Discussion

The purpose of this investigation was to characterize supermarkets, measure the distance from shoppers’ residence to supermarkets they shopped at, and explore the associations of these with shoppers’ BMI. We found that most shoppers lived more than one kilometre from the supermarket and that the price of a basket of common foods across all five supermarkets was inversely associated with BMI of its shoppers. This relationship between the price of food basket with BMI remained significant even after adjusting for socio-economic confounders. These findings suggest that many people shop for food outside of their immediate residential neighbourhood and that BMI may be a function of food pricing.

Investigations of the BE have predominantly focused on identifying the associations between individual characteristics such as lifestyle behaviours and obesity with characteristics in the surrounding environment, including potential destinations such as supermarkets. The main methods used for defining the extent of the environmental assessment include the use of existing geographical administrative boundaries such as census tracts and postal regions, or defining an area that encircles an individual’s place of residence, usually using a 500 m up to a 1500 m radius [19,20,22,24-26,29-31]. The acknowledged limitation of these methods is that it is based on the assumption people will frequently interact with BE features in their immediate neighbourhood. In our study we found that the minimum median distance between the destination supermarket and the shoppers’ residence ranged from 0.96 km to 4.30 km. Therefore, the majority of the participants surveyed shopped at a supermarket that was much farther away from their residence than the commonly used neighbourhood definitions in previous studies. The majority of participants also stated that the surveyed supermarket was their primary store for food shopping and that they frequented that supermarket more than once per week even though many of our participants lived actually closer to one of the other four supermarkets in our study than the one they shopped in and most study participants reported having the nearest produce store within five blocks or less of their home. While we calculated the minimum distance between each shoppers’ place of residence and the supermarket they frequented, it is not known if the participants came directly from home to the supermarket or in transit from another destination (i.e.: from work to home), which could reflect the convenience of shopping at that supermarket. Regardless, these findings suggest that the relationships between the BE and obesity are likely to be more complex than looking at an individual’s immediate neighbourhood. This complexity may also reflect the often reported low effect sizes and/or inconsistent results between the presence of neighbourhood supermarket with diet and obesity reported in earlier studies.

Of the different supermarket characteristics assessed, only the price of common food staples was associated with mean customer BMI such that as the price decreased, mean BMI increased. The associated increase was substantial such that BMI increased by 3.7 kg/m^2^ when comparing the supermarket with the lowest priced food basket to the highest. Empirically, it makes sense that as the price of consumer goods decreases, consumption increases, and there have been a number of advances in food production that has led to decreasing costs of foods which may be implicated in the population increase in BMI [32,33]. In addition, Courtemarche et al. found an association between the expansion of Walmart Supercenters and increasing BMI and obesity prevalence in the US and suggested this may be due, in part, to lower food prices [34]. While our food basket items may generally be labeled as consisting of somewhat healthier choices, it is possible that supermarkets that have lower prices in healthy foods may also have lower prices on less healthy foods. However, we do not know what exactly the participants purchased at the supermarket that day. Another possible explanation is that higher prices of healthier or staple food items may leave less money for more discretionary purchases of unhealthy foods or other items that may be implicated in obesity. A recent review by Epstein et al. indicated that in laboratory settings, purchase patterns of healthy and unhealthy foods did indeed change in concert with changing food prices such that purchases were inversely associated with price [35].

Our results are consistent with those recently reported by Drewnowski et al. [36] From their telephone-based survey, they reported no association between distance to the nearest supermarket and obesity but did observe a negative association between obesity and food pricing of supermarkets. The authors also observed that the participants who reported they shopped at the higher priced supermarkets had a higher household income and education than the participants who reported shopping at the lower priced supermarkets, and speculated that individuals with lower incomes shop at lower priced supermarkets regardless of proximity. However, the negative association between obesity and supermarket price remained even after adjusting for socio-economic status. This is similar to our results in that the adjustment for area median individual income and personal car ownership did not affect the relationship between supermarket ‘food basket’ price and BMI of supermarket shoppers. Therefore, it is unlikely that individual income and education alone can explain the negative association between food pricing and obesity.

With the increase in obesity, a number of studies have begun to investigate interventions in which food prices have been altered to affect food purchasing habits, either through decreasing pricing of healthier foods and/or increasing pricing of unhealthy foods. In the laboratory setting, studies have shown promise in influencing consumer habits, however, the findings are less clear in the real world setting [35]. In a recent, well-designed, randomized control trial where the intervention consisted of food price discounts on healthy foods in eight supermarkets in New Zealand, Mhurchu et al. found no differences in overall nutrients compared to those participants without the discounts; however, the amount of healthier foods purchased was greater at 6 months (end of intervention) and 12 months, although there was little change in the less healthier food purchases [37]. This is consistent with reviews that have reported that taxes and/or subsidies generally resulted in the desired changes in food consumption patterns and this increased as the tax increased, however, the evidence is less clear on the effects on BMI [38,39]. This may be due to the interventions being too short duration to make a lasting and sustainable change in BMI, and that longer interventions are needed.

### Limitations

Our findings are limited by the cross-sectional nature of the investigation, and we cannot discern conclusively whether the lower food prices are implicated in obesity or whether obese individuals preferentially shop at lower priced supermarkets, nor whether the food basket price was a marker for some other causal factor. Despite this, our findings do provide insight into these associations that can form the foundation for directing interventions. Another limitation is the use of self-reported height and weight, which has been reported to underestimate BMI [40]. However, this error in self-reporting is unlikely to alter the associations we observed. We also did not have information regarding the actual food items purchased at the surveyed supermarket, nor participant dietary intake.

## Conclusions

The current investigation has two key findings and implications. One that the overwhelming majority of shoppers surveyed lived more than one kilometre from the supermarket they shopped at, and many lived farther than two kilometres away. This finding has implications regarding the appropriateness of exploring only the built food environment characteristics, such as supermarkets, that are closest to a participant’s place of residence or the ones positioned within neighbourhoods defined as administrative units or circular zones around individuals. Such methodology may lead to erroneous conclusions about the BE and may in part explain the small effect sizes that the presence of food outlets have with respect to associations with obesity. Investigating the associations between the characteristics of food outlets with the customers will give a greater insight on the mechanisms behind the associations between built food environment and obesity. The other key finding is that the price of a collection of food staples of the supermarket was inversely associated with the shopper’s BMI. These findings persisted even after taking into account demographic and socio-economic indicators. While increasing food prices may have unintended, and harmful, consequences, our results suggest that careful manipulation of food prices may indeed be a point of intervention for addressing the increase in obesity. Further research should focus on the actual interactions individuals have with the environment and also take into account the internal aspects of these features so that health promotion strategies can be robustly informed.

## Competing interests

The authors declare that they have no competing interests.

## Authors’ contributions

SAL designed the study, performed statistical analyses and wrote the manuscript. DG participated in the interpretation of the data and helped to draft the manuscript. NS carried out and interpreted GIS-related analyses. All authors read and approved the final manuscript.
